# Annotations

**Published:** 1909-09-11

**Authors:** 


### ANNOTATIONS.

### Rate Aid for the Hospitals.

Last week Mr. J. MacVeagh introduced into the House of Commons the following Bill to empower local authorities to strike a rate in aid of local hospitals:—" Be it enacted by the King's most excellent Majesty, by and with the advice and consent of the Lords Spiritual and Temporal, and Commons,, in this present Parliament assembled, and by the authority of the same, as follows: (1) From and after *the passing of this Act* it shall be lawful for any district council, parish council, corporation, board of town commissioners, county council, or other rating authority tc strike a rate not exceeding *threepence in the pound* on the valuation towards the erection or maintenance of any hospitals situated within the area of charge. (2) The allocation of any rates so levied shall be subject in England and Wales to the approval of the Local Government Board, in Ireland to the approval of the Local Government Board for Ireland, and in Scotland to the approval of the Local Government Board for Scotland. (3) This Act may be cited as the Hospitals Act, 1909." It is a private Bill, and certainly not of the class which can be passed without discussion or debate. There is, therefore, little chance that it will reach a second reading this session in view of the press of urgent business with which the House of Commons has still to deal. That is, of course, fully understood by its sponsor, who states that his main object in bringing forward the Bill at this late stage is to draw public attention to its terms and to elicit extra mural discussion on the subject with which it deals. The measure is an important and grave step which should be carefully considered by everyone who is interested in institutional matters. We hope to return to its discussion on a future occasion.

### The Treatment of Leprosy.

In an interesting report Dr. Ernest Moon, senior medical officer of the Leper Settlement at Robben Island, devotes some attention to the results of new lines of treatment which have been tried on patients under his charge. In addition to the internal and external use of chaulmoogra oil which has been used in the past year, and under which some of the patients, who take an interest in their case and persevere with the treatment, show an improvement, several of these patients are also taking one-fiftieth of a grain of strychnine twice daily, with benefit to themselves. Three male Europeans and four female Europeans are on Dr. Duque's treatment of Havanna, consisting of liquid extract of red mangrove bark, commencing with 7 c.c. (about two drachms) twice daily after meals, and increased weekly by double doses till 80 c.c. (about 3 ozs.) are reached. They are also having hot baths, about 100 degrees, the last thing at night. The bath water is discoloured with the mangrove. Dr. Moon concludes: "The treatment was commenced in April and May months. I regret I cannot see any improvement in their condition. The medical officer of health for the colony sent some roots which had been given to him by Mr. J. W. Jagger, M.L.A., consequently called "Jagger's Roots." These when powdered were to be applied locally to the active maculæ in anæsthetic cases. The nature of the roots is unknown to me, but I understand they were given by a friend to Mr. Jagger with a history that they were used by the natives of Rhodesia for the cure of leprosy. They were said to convert active maculæ into white spots. In two cases the extract was tried without any favourable result."

### Teething Powders.

Even among the educated classes that almost ineradicable maternal instinct to ascribe all and every infantile disorder to some fanciful and often extremely remote cause dies very hard. Among the labouring population it is not surprising therefore that enlightenment should be perceptibly slower still in making headway. Of all the processes which have thus to bear the supposititious burden of creating ills innumerable, that of teething stands easily first in popular imagination, outdistancing vaccination by a great distance. There is probably no single malady that may afflict an infant which is not set down by mothers (and grandmothers) to the occurrence of dentition. Since the eruption of teeth continues more or less regularly from the age of six months to that of two years, it follows that there can never be any moment between those ages when a tooth has not either lately been cut or shown signs of being about to be so. Herein, naturally, lies much of the strength of the superstition in question, for it is nothing less. Since this idea is so universal, it is not surprising that the public should seek remedies for the dangerous epoch of tooth-eruption; and the number of "teething powders" on the market and their ready sale are evidence of the faith with which this delusion is still cherished. If such quack compounds were always harmless, little evil might result beyond the swindling of gullible mothers by unscrupulous nostrum vendors: but unfortunately this is far from being the case. It appears from the reports of two inquests held about the end of August at Southwark that a mother administered a teething powder "to cool the blood" of her infant son, then suffering from diarrhœa. Whether this particular powder contained opium—as many do—or grey powder or calomel—ingredients equally common—does not appear; but the baby became rapidly worse, and died shortly afterwards. Another child, which had been similarly treated, died whilst being taken to Guy's Hospital. The coroner (Dr. Waldo) took occasion to point out that the patent medicine duty stamp on these secret remedies is in no sense a guarantee of their worth. It seems extraordinary that this idea should be so widely diffused, in spite of frequent efforts to expose its absurdity, and in spite of the plain statement to the contrary on the stamp itself; but in fact the semi-literate proletariat do entertain it very widely, and no effort to counteract it is superfluous. He also elicited from medical witnesses the familiar fact that many of these mercurial remedies, when kept long in stock, oxidise slowly into forms which generate corrosive sublimate on entering the stomach.

